# Assessment of the core and support functions of the integrated disease surveillance and response system in Zanzibar, Tanzania

**DOI:** 10.1186/s12889-021-10758-0

**Published:** 2021-04-17

**Authors:** Fatma Saleh, Jovin Kitau, Flemming Konradsen, Leonard E. G. Mboera, Karin L. Schiøler

**Affiliations:** 1 Department of Parasitology and Entomology, Kilimanjaro Christian Medical University College, Moshi, Tanzania; 2 Department of Allied Health Sciences, School of Health and Medical Sciences, The State University of Zanzibar, Zanzibar, Tanzania; 3 World Health Organization, Country office, Dar es Salaam, Tanzania; 4 Global Health Section, Department of Public Health, University of Copenhagen, Copenhagen, Denmark; 5 SACIDS Foundation for One Health, Sokoine University of Agriculture, Morogoro, Tanzania

**Keywords:** Disease surveillance, Core, Support, Functions, Outbreaks, Zanzibar

## Abstract

**Background:**

Disease surveillance is a cornerstone of outbreak detection and control. Evaluation of a disease surveillance system is important to ensure its performance over time. The aim of this study was to assess the performance of the core and support functions of the Zanzibar integrated disease surveillance and response (IDSR) system to determine its capacity for early detection of and response to infectious disease outbreaks.

**Methods:**

This cross-sectional descriptive study involved 10 districts of Zanzibar and 45 public and private health facilities. A mixed-methods approach was used to collect data. This included document review, observations and interviews with surveillance personnel using a modified World Health Organization generic questionnaire for assessing national disease surveillance systems.

**Results:**

The performance of the IDSR system in Zanzibar was suboptimal particularly with respect to early detection of epidemics. Weak laboratory capacity at all levels greatly hampered detection and confirmation of cases and outbreaks. None of the health facilities or laboratories could confirm all priority infectious diseases outlined in the Zanzibar IDSR guidelines. Data reporting was weakest at facility level, while data analysis was inadequate at all levels (facility, district and national). The performance of epidemic preparedness and response was generally unsatisfactory despite availability of rapid response teams and budget lines for epidemics in each district. The support functions (supervision, training, laboratory, communication and coordination, human resources, logistic support) were inadequate particularly at the facility level.

**Conclusions:**

The IDSR system in Zanzibar is weak and inadequate for early detection and response to infectious disease epidemics. The performance of both core and support functions are hampered by several factors including inadequate human and material resources as well as lack of motivation for IDSR implementation within the healthcare delivery system. In the face of emerging epidemics, strengthening of the IDSR system, including allocation of adequate resources, should be a priority in order to safeguard human health and economic stability across the archipelago of Zanzibar.

## Background

The Integrated Disease Surveillance and Response (IDSR) strategy was adopted by the World Health Organization Regional Office for Africa (WHO-AFRO) during its 48th Assembly in 1998, as a means towards strengthening epidemiologic surveillance and response in the African region [1, 2]. Evaluation of IDSR systems in Sub-Saharan Africa have identified some successes on its implementation including increased national level use of surveillance data, improved communication and coordination between districts and other sectors as well as the availability of IDSR reports through district health information systems (DHIS) [2–7].

Notably, significant shortfalls have also been reported across the region, particularly on outbreak preparedness, timeliness of IDSR reports, quality of reported data, inadequate laboratory networks, lack of an effective IDSR strategy at community level as well as provision of regular feedback and supervision [2, 3, 5–7]. Other factors include inadequate financial resources, inadequate training and high turnover of peripheral staff, and poor communication and transport systems particularly at the periphery [8].

**Fig. 1:**
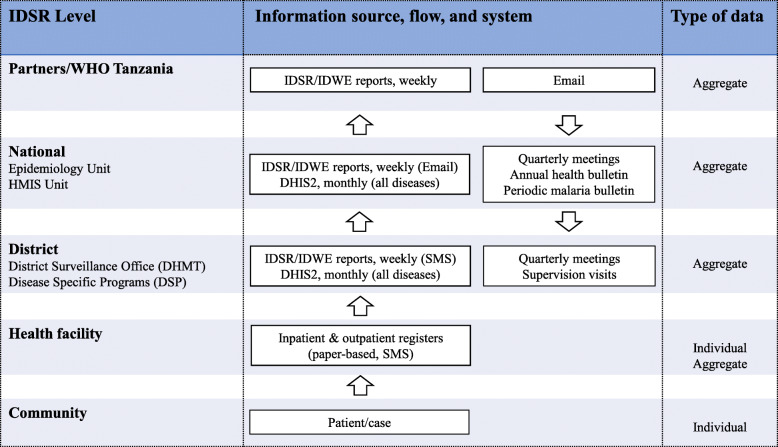
Organization of Zanzibar IDSR system showing the flow of information and feedback. Source: Adapted from Joseph Wu et al. [10]. IDWE: Infectious Disease Week Ending. HMIS: Health Management Information System. DHMT: District Health Management Team. DSP: Malaria, TB & Leprosy, HIV/AIDS

Zanzibar adopted the IDSR strategy in 2010 when national guidelines were developed by the Ministry of Health [9] following WHO guidelines [1]. Accordingly, the strategy was introduced at each level of the Zanzibar healthcare delivery system (national, district, and health facility) where all health facilities, public and private irrespective of level were required to submit disease surveillance reports to their respective districts and eventually to the national Ministry [9] (Fig. 1). The priority communicable diseases required to be reported weekly include malaria, cholera, bloody diarrhoea, diarrhoea, measles, yellow fever, dengue, viral haemorrhagic fevers, chikungunya, plague, rabies, human influenza, typhoid, pneumonia, chickenpox, smallpox, anthrax, keratoconjuctivitis, cerebrospinal meningitis, acute flaccid paralysis, rabies, neonatal tetanus, and trypanosomiasis [9].

Although Zanzibar operates at a much smaller scale, it is likely to face similar challenges to that of other African countries in sustaining an effective IDSR system. These challenges are of particular concern in the case of infectious diseases, for which incipient epidemic activity may be difficult to monitor, hampering timely detection, and response. Despite its long-term implementation, the performance of the Zanzibar IDSR system has yet to be assessed. This study was carried out to assess the performance of the core and support functions of Zanzibar IDSR system to determine its capacity for early detection of and response to infectious disease outbreaks.

## Methods

### Study setting

Zanzibar archipelago is situated off the coast of East Africa. It recorded a population of 1,303,569 and an annual growth rate of 3% in the 2012 population census [11]. The majority (70%) of the population lives on the island of Unguja and 30% on Pemba Island [11]. At the time of the study, Zanzibar had 10 districts each with a corresponding District Health Management Team (DHMT) which serves as a link between health facilities and national level and is responsible for organizing and reporting of all health data from health facilities to the central ministry [9]. The public health infrastructure was organized into 152 primary health care units (PHCUs) and four primary health care centres (PHCCs), two district hospitals, two specialized hospitals (maternity and mental health), one regional, and one national referral hospital. In addition, there were 90 privately owned or managed health facilities including dispensaries/clinics and hospitals across the archipelago [12].

### Study design

This cross-sectional descriptive study evaluated the core and support functions of the Zanzibar IDSR system. The core surveillance functions include case detection, registration and confirmation, reporting, data analysis, epidemic preparedness and response, and provision of feedback. The support functions include availability of standard guidelines for surveillance, staff training, supervision, communication facilities, laboratory capacity, availability of resources, and coordination.

### Sampling

Health facilities were sampled across the archipelago, with representation of all strata including administrative areas (districts), rural and urban settings, as well as public and private facilities [13]. Public and private health facilities were selected by stratified random sampling using each of the 10 districts as strata. In each district, PHCUs were selected using simple random sampling while all PHCCs and non-specialized hospitals were included, as were each District Health Management Team (DHMT) and the Epidemiology and HMIS units at the central Ministry of health.

Forty-five health facilities; 30 from Unguja and 15 from Pemba, were included. Of these, five were hospitals, four PHCCs and 36 PHCUs. Among the included facilities on Unguja Island, 20 were public and 10 privately owned, whereas in Pemba, 13 were public and two were private.

### Data collection

The researcher and a team of three trained assistants collected all data in November and December 2017. The collected data involved the IDSR reporting period of July to September 2017. The study participants included 45 health facility in-charges or designated disease surveillance staff (one person per facility), 10 district surveillance officers (DSOs) (one person per district) and two staff from the Epidemiology and Health Management Information System (HMIS) units (one person per unit). The latter two were included to capture the flow and utilization of health data (Fig. 1). The evaluation was conducted following a WHO guide [14] and protocol [13] for assessment of national communicable disease surveillance and response systems. Structured interviews were completed using questionnaires adapted from the WHO protocol [13]. The modified questionnaires included open and closed-ended questions validated at a stakeholder’s workshop at the Ministry of Health prior to data collection. During interviews, direct observations were carried out to verify the availability of standard case definitions, reporting tools and guidelines, as well as evidence of data analysis (visible graphs, charts). A review of secondary data included weekly and monthly surveillance reports, patient case registers, standard case definitions, data analysis results, feedback bulletins, supervision plans and checklists, as well as minutes of surveillance related meetings wherever available.

Qualitative data were collected from open-ended questions relating to IDSR implementation challenges and strategies for improvement through interviews with key surveillance staff at central, district and health facility levels.

### Data analysis

Descriptive analysis of questionnaire data was completed in SPSS for Windows (version 20.0, Armonk, NY). Frequency distribution tables were prepared, and proportions calculated based on specific performance evaluation indicators stratified by district and health facility levels. The narrative responses arising from open-ended questions relating to IDSR implementation challenges and strategies for improvement were thematically analysed.

## Results

### Core surveillance functions

Table 1 provides an overview of the performance of the six core surveillance functions of the Zanzibar IDSR system at the health facility (public and private) and district levels.

**Table 1:** Performance of the IDSR core surveillance functions at health facility and district levels, Zanzibar

Core activity	Primary health care facilities^a^		Hospitals		
	Public^**b**^ (***n*** = 29)	Private^**c**^ (***n*** = 11)	Public^b^ (***n*** = 4) + Private^c^ (***n*** = 1)	Facility Total (***n*** = 45)	District Total (***n*** = 10)
	n (%)	n (%)	n (%)	n (%)	n (%)
Case Detection and Registration					
Availability of standard case definitions for each priority diseases	21 (72)	2 (18)	0 (0)	23 (51)	NA^d^
Correctly diagnosed at least one priority disease	24 (83)	9 (82)	2 (40)	35 (78)	NA
Availability of outpatient clinical register	29 (100)	10 (91)	5 (100)	44 (98)	NA
Case Confirmation					
Capacity to handle specimens until shipment	23 (79)	7 (64)	4 (80)	34 (76)	NA
Capacity to transport specimens to higher level laboratory	10 (35)	3 (27)	3 (60)	16 (36)	10 (100)
Availability of guidelines for specimen collection, handling, and transportation	11 (38)	8 (73)	3 (60)	22 (49)	5 (50)
Data Reporting					
Availability of adequate supply of surveillance forms in the past 6 months	26 (90)	9 (82)	5 (100)	40 (89)	8 (80)
Availability of a formalized system for reporting to the next level					
For weekly report	0 (0)	0 (0)	0 (0)	0 (0)	0 (0)
For monthly report	0 (0)	0 (0)	0 (0)	0 (0)	10 (100) via DHIS2^e^
Data Analysis					
Analyse and present data by person	6 (21)	0 (0)	0 (0)	6 (13)	3 (30)
Analyse and present data by place	4 (14)	0 (0)	0 (0)	4 (9)	1 (10)
Analyse and present data by time	4 (14)	0 (0)	0 (0)	4 (9)	2 (20)
Perform trend analysis	3 (10)	0 (0)	0 (0)	3 (7)	2 (20)
Epidemic Preparedness and Response					
Availability of epidemic preparedness and response plan	NA	NA	NA	–	6 (60)
Availability of budget line for epidemic response	NA	NA	NA	–	10 (100)
Have rapid response team for epidemics	NA	NA	NA	–	10 (100)
Availability of action threshold for the country’s priority diseases	21 (72)	5 (45)	1 (20)	27 (60)	10 (100)
Availability of standard case management manual for epidemic prone diseases	20 (69)	6 (55)	1 (20)	27 (60)	NA
Availability of emergency stocks of drugs/supplies in past 1 year	NA	NA	NA	–	8 (80)
Experienced shortage of drugs/supplies during the most recent outbreak	NA	NA	NA	–	5 (50)
Feedback					
Received written feedback report/bulletin from higher level	7 (24)	1 (9)	0 (0)	8 (18)	5 (50)
Produced written feedback reports in the last year	NA	NA	NA	–	1 (10)

^a^Primary health care units (PHCUs) and primary health care centres (PHCCs). *PHCU* first level health care facility providing basic primary health care services within specified daytime hours. *PHCC* second level primary health care facility providing services 24 h a day [12]. ^b^Publicly owned. ^c^Privately owned. ^d^*NA* Not applicable. ^e^*DHIS2* District Health Information Software 2

### Case detection and confirmation

Through direct observations, we identified the availability of standard case definitions (SCDs) for the priority diseases in 23 of 40 (57%) primary health facilities including 21 of 29 (72%) public and 2 of 11 (18%) private. None of the hospitals had SCDs in place. However, almost 80% (35/45) of the interviewed health facility staff could correctly diagnose at least one priority disease according to SCD, most frequently cholera.

The capacity of health facilities to diagnose or confirm priority diseases was generally low, as most facilities particularly PHCUs lacked appropriate laboratory equipment and supplies needed for specimen collection or testing. About two thirds (31/45, 69%) of the health facilities had the capacity to collect blood or serum samples, while five (11%) health facilities had the capacity to collect cerebrospinal fluid samples. Sputum for Mycobacterium examination and stool specimens for microscopy could be sampled in 25 of 45 (55%) and 18 (40%) of the health facilities, respectively. For most facilities, the capacity to test the collected samples were limited to malaria testing. In general, hospitals had higher capacity for specimen testing where all 5 (100%), in addition to malaria testing, could perform other tests including serology, microbial culture, and antibiotic sensitivity tests.

About three-quarters (34/45, 76%) of the facilities had the capacity to handle specimens until shipment, as they had a functional cold chain in place. However, the capacity to transport specimens to referral laboratories was minimal due to the absence of appropriate materials for packaging and transportation. Notably, half of the facilities and districts did not have guidelines for specimen collection, handling, and transportation (Table 1). As such, almost all facilities referred patients rather than specimens, leaving the patients or their relatives with the responsibility of acquiring the prescribed tests, causing delays in diagnoses and treatment.

### Data reporting

In terms of data reporting, nearly 90% (40/45) of health facilities and 80% (8/10) of districts reported having an adequate supply of surveillance forms during the previous 6 months. However, the monthly disease summary forms and case investigation forms were occasionally missing. Notably, reporting to the next level presented a substantial challenge at facility level. In the absence of a computerized system, staff reported using phone text messages to submit weekly infectious disease/IDSR report to the district office. In addition, monthly (all diseases) paper-based surveillance forms were hand-delivered to the district offices often using public transport as there were no direct means of transport. Alternatively, staff waited for the DSO to collect the forms during supervision visits, which often caused delays in submission. In contrast, all districts used the District Health Information Software 2 (DHIS2), a formalized electronic database, for submitting the monthly reports to the HMIS unit at the Ministry of Health. For the weekly IDSR reports however, as in the case of health facilities, no computerized system existed at district level, thus reports were sent directly to the Ministry Epidemiology unit via phone text messages. This has often led to delay or non-submission as reported by district surveillance staff. When interviewed, health facility staff and DSOs reported lack of airtime voucher, poor telephone networks and lack of incentives for reporting as reasons for delay or non-submission of data.

### Data analysis

Through observation and document reviews, it was revealed that data analysis was rarely practiced at facility and district levels with most lacking visible line graphs for any of the priority diseases. Only three of 29 (10%) public primary health facilities carried out their own analysis, mostly for malaria, diarrhoea, cholera, or pulmonary tuberculosis. None of the private facilities or hospitals conducted data analysis. Even at national level, data analysis was not regularly conducted except during outbreaks when trend analysis was done for the particular disease. Despite the presence of appropriate denominators at all district and national levels, epidemiological parameters including incidence, prevalence and case fatality were rarely calculated. No calculated rates were observed during the assessment period except for three out of 10 (30%) districts where case fatality rates were computed for cholera.

### Epidemic preparedness and response

All districts had action thresholds for priority diseases as opposed to 60% (27/45) of the facilities. Diseases for which action thresholds were mostly available were cholera, malaria, measles, tuberculosis, acute flaccid paralysis, rabies, dengue, and yellow fever. Forty percent of the health facilities did not have a written standard case management protocol for any of the epidemic prone diseases. Where available, the most commonly found manuals were for cholera and malaria.

All 10 districts reported to have outbreak rapid response teams and budget lines specifically allocated for epidemic response. However, only 60% (6/10) had developed their epidemic preparedness and response plans. While 70% (7/10) of the districts reported to have epidemic management committees, these were all reported to be inactive and did not have plans for regular meetings. Furthermore, their preparedness and response activities had not been evaluated within the past year.

### Feedback

None of the facilities or districts reported receiving feedback reports on a regular basis. Only 8 of 40 (20%) primary health facilities had received a written feedback report or bulletin from higher levels in the past year and in all instances, it was on malaria. None of the hospitals had received any kind of written feedback report. Only half of the districts received written feedback from the national level in the form of an annual health bulletin. Notably, no epidemiological bulletin or district newsletter were observed during the assessment. Only one out of 10 (10%) district produced information summary sheets on a quarterly basis. Public facilities (23/33, 69%) were more likely than private facilities (4/12, 33%) to receive verbal feedback during quarterly meetings. However, these meetings were reported to be irregularly planned and conducted. Three of 29 (10%) public primary facilities reported receiving feedback during supervision visits conducted by DHMT and occasionally by the Epidemiology Unit. In addition, 7% (3/45) of the health facilities received occasional telephone calls in case of data discrepancy or a suspected outbreak.

## Support surveillance functions

### Availability of standard guidelines, and supervision

The national IDSR guidelines developed in 2010 were available in seven out of 10 districts (70%), 4 primary health facilities (public = 3; private = 1) and none of the hospitals. Likewise, there was no clear guidelines or plan on the required number of supervisory visits at any district or national level. Nevertheless, health facilities were more likely to receive supervision as compared to the districts. Almost all public primary health facilities (28/29, 97%) and a majority (8/11, 73%) of the private primary health facilities reported at least one supervision visit by the district surveillance staff within the last 6 months. Only one out of five hospitals (20%) reported to have received at least one supervision visit during the past 12 months. Furthermore, only 40% (4/10) of the districts received supervision by national level surveillance staff. The most common explanation offered for the inadequate supervision were high staff workloads at districts (40%), inadequate transport facilities including fuel shortage (60%), and lack of vehicle at the national level surveillance unit.

### Training, and coordination

There were no surveillance training plans or database of trained surveillance personnel at the national or district levels. Only the national surveillance focal person had a post-graduate degree in field epidemiology with extensive training on disease surveillance. Training on disease surveillance was much higher at district than facility level. All district surveillance officers had received basic training on disease surveillance either in the form of short (2 days – 1 week) in-service trainings (8/10, 80%) or as part of their degree programs (2/10, 20%). In contrast, surveillance staff at half of the health facilities had no training on disease surveillance, and most of those trained received only 1 to 3-day course. Surveillance personnel in public primary facilities (18/29, 62%) were more likely to be trained than those in private primary facilities (4/11, 36%) or hospitals (2/5, 40%).

The staff knowledge on the IDSR strategy was generally very low across health facilities and districts. Only 3 of 10 (30%) district surveillance staff and 5 of 29 (17%) public primary health facility staff were able to describe the IDSR strategy, correctly. The strategy was still understood as merely a system of collection and reporting of information on diseases (40% of districts) or a system of reporting infectious and notifiable diseases (30% of districts). Likewise, awareness on level-specific IDSR indicators was very low at all levels (Table 2).

**Table 2:** Performance of the IDSR support surveillance functions at health facility and district levels, Zanzibar

Support activity	Primary health care facilities^a^		Hospitals		
	Public^**b**^ (***n*** = 29)	Private^**c**^ (***n*** = 11)	Public^**b**^ (***n*** = 4) + private ^**c**^ (***n*** = 1)	Facility Total (***n*** = 45)	District Total (***n*** = 10)
	n (%)	n (%)	n (%)	n (%)	n (%)
Standards and guidelines for surveillance					
Availability of national guidelines for surveillance	3 (10)	1 (9)	0 (0)	4 (9)	7 (70)
Supervision					
Supervised by higher level supervisor in the last 6 months	28 (97)	8 (73)	1 (20)	37 (82)	4 (40)
Supervised health facility staff	NA^d^	NA	NA	–	7 (70)
Have supervision checklist	NA	NA	NA	–	6 (60)
Training					
Knowledge on IDSR	5 (17)	0 (0)	0 (0)	5 (11)	2 (20)
Awareness on IDSR indicators	2 (7)	0 (0)	0 (0)	2 (4)	2 (20)
Trained on disease surveillance	18 (62)	4 (36)	2 (40)	22 (49)	10 (100)
Coordination					
Availability of surveillance focal person at the district	NA	NA	NA	–	10 (100)

^a^Primary health care units (PHCUs) + primary health care centres (PHCCs). ^b^Publicly owned. ^c^Privately owned. ^d^*NA* Not applicable

In terms of coordination, each district had a focal person for surveillance activities who also served as a surveillance focal point within the district epidemic management committee.

### Laboratory capacity

The laboratory capacity was generally inadequate at all health facility levels. About half (19/36) of the PHCUs did not have capacity to collect or test any kind of sample, either due to lack of laboratory facilities (equipment and materials) or personnel. The remaining PHCUs (*n* = 17) could only carry out microscopic examination of blood smear for malaria parasites. In case of other tests, the patients were referred to higher-level facilities or private laboratories if available. At the hospital level, mostly culture and antibiotic sensitivity, and serological tests were performed. The reference laboratory at Mnazi Mmoja Hospital in Unguja and Public Health Laboratory in Pemba did not have the capacity to carry out molecular diagnosis for any of the priority diseases. Blood samples from suspected cases of infectious disease requiring molecular diagnosis for confirmation were shipped to the national laboratory in mainland Tanzania, a transfer of minimum four hours. On average, Zanzibar Ministry of Health would receive the molecular test results in 48 h.

### Resources for surveillance

Resources for surveillance activities were more often available at district level compared to facility level. Each district surveillance office had almost all resources required for data management including a designated data manager, a functional computer, printer, and stationeries. The basic information and communication materials as well as logistic support including transportation were also available in most districts. Nonetheless, shortage of fuel was often reported as a major challenge hampering regular and adequate supervisory visits at district and national levels. In addition, despite availability of a budget line for surveillance, inadequate financial resources were frequently reported at district and national levels as a major reason for not conducting regular trainings. In addition, availability of communication resources was a major issue in almost all districts. At facility level, all resources were suboptimal (Table 3).

**Table 3:** Available resources for IDSR at health facility and district surveillance offices, Zanzibar

Resource	Public facility (*N* = 33)	Private facility (*N* = 12)	Facility Total (*N* = 45)	District Total (*N* = 10)
	n (%)	n (%)	n (%)	n (%)
Data Management				
Data Manager	10 (30)	7 (58)	17 (38)	10 (100)
Computer	12 (36)	8 (67)	20 (44)	10 (100)
Printer	10 (30)	4 (33)	14 (31)	10 (100)
Photocopier	5 (15)	4 (33)	9 (20)	7 (70)
Stationery	17 (52)	2 (17)	19 (42)	10 (100)
Statistical package	1 (3)	1 (8)	2 (4)	2 (20)
Communications				
Telephone service	14 (42)	8 (67)	22 (49)	2 (20)
Computer with internet modem	6 (18)	4 (33)	10 (22)	4 (40)
IEC^1^ Materials				
Posters	29 (88)	7 (58)	36 (80)	10 (100)
Flip Charts	21 (64)	1 (8)	22 (49)	10 (100)
Projector	5 (15)	1 (8)	6 (13)	5 (50)
Logistics				
Reliable electricity	32 (97)	12 (100)	44 (98)	10 (100)
Bicycle	5 (15)	0 (0)	5 (11)	2 (20)
Motorcycle	2 (6)	0 (0)	2 (4)	8 (80)
Vehicle	8 (24)	2 (17)	10 (22)	8 (80)

^1^*IEC* information, education, and communication

### Overall IDSR implementation challenges

The interviewed surveillance personnel at different levels of the healthcare delivery system expressed different challenges on the implementation of the IDSR strategy, while some weaknesses were common to all levels. The key themes/challenges identified from the narrative responses to unstructured interview questions are illustrated in Table 4 by healthcare system level. Lack of electronic system for reporting weekly surveillance data, low staff knowledge/training, and financial constraints were common to all levels as exemplified:

“*… .implementation of the IDSR strategy in general is mostly affected by lack of an electronic system, and inadequate financial resources that affect provision of regular trainings and supervision of the district and health facility staff ….*” . (national level surveillance staff).

*“… .it is like the IDSR system does not exist, because unlike HMIS where we use DHIS2 for routine monthly reporting, there is no computerized system for reporting infectious disease week ending (IDWE) reports … .and the use of short message service (SMS) have proved ineffective because it is often associated with delay or failure to report particularly at health facility level. Sometimes we have to call them (health facility staff) several times to remind them to submit reports”. (district surveillance staff).*

*“… .there is no proper system for report submission, we submit the weekly IDWE reports to the district office through SMS, and routine monthly reports in paper-based forms. This is very cumbersome, costly, and time consuming … …*. *and we are not regularly supplied with airtime vouchers or anything to support this activity ….”.* (health facility surveillance staff).

“*… .there is no training, and we are not regularly supervised to see if we are using the reporting tools correctly*”. (staff at a private health facility).

**Table 4:** Zanzibar IDSR implementation challenges by the healthcare delivery system level

Level	Expressed challenges
**National**	Lack of electronic system/database for infectious disease reporting
	Inadequate financial resources for conducting regular supervision visits and training
	Lack of back-up system for data security
**District**	Lack of electronic system for infectious disease reporting
	Inadequate resources including fund for conducting supervision visits
	Low staff knowledge on IDSR strategy particularly at health facilities
	Delay of reports from health facilities particularly privately owned
	Poor communication system for reporting suspected outbreaks
	Unreliable internet service
	Lack of incentives for IDSR reporting leading to low staff motivation
**Health facility**	High staff workload
	Filling paper-based surveillance forms time consuming
	Late report collection by DSOs
	Absence of airtime vouchers for submitting weekly cell phone-texted data
	Lack of regular trainings or capacity building on disease surveillance and IDSR reporting tools
	Inadequate supervision and feedback from higher levels
	Lack of designated personnel responsible for IDSR at the hospital level

### Strategies for improving surveillance

Through unstructured interview questions, the key IDSR personnel at the national, district and health facility levels in general recommended the following strategies for improving disease surveillance: establishment of an electronic database for weekly IDSR reporting, provision of regular trainings for surveillance staff at all levels, provision of adequate resources for IDSR implementation, regular follow up and supervision, and feedback from higher levels. Others included the need for incorporating IDSR training into health curricula, provision of incentives, and sensitization of staff on the importance of IDSR reporting. The following are some of the solutions for improving surveillance narrated by surveillance staff:

*“… staff are not motivated to participate in IDSR, … to improve surveillance, staff should be sensitized to understand the importance of reporting and should be given incentives to motivate them … … .and the Ministry should introduce electronic system for IDSR reporting from health facilities onward”*. (district surveillance staff).

*“… .regular trainings should be conducted, …. we should be given airtime vouchers for submitting weekly reports, and DSOs should supervise and give us feedback on the reports we submit”.* (health facility staff).

## Discussion

This evaluation provides an important insight on the performance of both core and support functions of the IDSR system in Zanzibar, while highlighting its capacity on early detection of and response to infectious disease epidemics. Our findings show that, despite notable achievements in integration of surveillance activities, geographical representativeness of the system with inclusion of both public and private health facilities in disease reporting, and allocated surveillance staff at all levels, the performance of the IDSR system in Zanzibar is generally unsatisfactory. Nearly a decade after its implementation, significant gaps remain to be addressed to optimize the system’s performance for early detection and response to epidemics. Similar gaps have been reported in other countries in sub-Saharan Africa owing to the same conditions including lack of sustainable resources and trainings, weak laboratory capacity, poor coordination, communication, and transportation services, and low staff motivation [10, 15–18].

The use of SCDs is the vital first step for a health provider to detect a case or an outbreak [14]. However, SCDs were infrequently applied particularly at hospital level leaving clinicians to rely on their knowledge and experience in making diagnoses with risk of missing cases or incipient outbreaks. Most health facilities in sub-Saharan Africa lack capacities to detect diseases through laboratory confirmation and health providers often rely on clinical manifestations to diagnose suspected cases [17], underscoring the need for SCDs to be made available at all times and all levels of the health care system. Studies in other African settings have demonstrated the importance of distribution and use of job aids including validated SCDs and surveillance guidelines in strengthening disease surveillance [4, 5, 17, 19].

Case confirmation was the poorest performing IDSR core function. Weak laboratory capacity at all levels of the healthcare delivery system greatly hampers investigation and confirmation of priority health conditions. Most health facilities particularly the PHCUs, which in most cases serve as first patient contact, lacked appropriate laboratory equipment and supplies needed for testing and confirmation of priority diseases in line with reports from other countries in sub-Saharan Africa and India [2, 20–22]. Notably, none of the health facilities or laboratories in Zanzibar could confirm all the priority diseases either due to lack of equipment and materials or skilled personnel. Reliance on shipment of samples to the national laboratory in mainland Tanzania for molecular confirmation of suspected epidemic diseases, require urgent attention as this may jeopardize timely response. To alleviate the diagnostic challenges in primary health care settings, the use of rapid diagnostic tests has been suggested [20]. In addition, development of capacity in terms of laboratory personnel and equipment, establishment of functional laboratory networks and framework of coordination between the laboratories and disease surveillance units at all levels are crucial for IDSR [2, 21]. The small geographical scope of Zanzibar offers possibilities for collaborations among these entities. Notably, the recently introduced molecular diagnostic services at Zanzibar National Referral Hospital, currently used for testing COVID-19 suspect cases and overseas travellers, provide an opportunity for extending the services to other epidemic-prone diseases which require molecular confirmation, given appropriate supplies and technical support.

Currently, all health facilities in Zanzibar submit paper-based forms to the district office with the volume of data overburdening the staff as also reported in other studies in Malawi, India, and Tanzania [10, 20, 23]. Whereas short message service (SMS) have been reported as a useful tool for reporting weekly infectious diseases in Uganda, Tanzania, and Central African Republic [18, 23, 24], the use of SMS in Zanzibar was associated with both delay and failure to report, which is in line with a study in Madagascar where text message transfer hampered timeliness and quality of data [25]. The delay and incomplete submission of reports coupled with lack of data analysis, data audit, and inadequate coordination and communication mechanisms could have devastating consequences in the event of epidemic development [26].

Introduction of a computerized system can allow real-time surveillance, simplify reporting and improve timeliness, completeness and quality of data hence strengthens system’s performance as seen in Kenya, Sierra Leone and elsewhere [26–29]. The electronic DHIS2 currently available at district level provide an opportunity to leverage an extension to peripheral levels if all health facilities receive appropriate and sufficient equipment, technical and logistic support.

Data analysis was found to be very weak at all levels as observed in other studies in Tanzania, Uganda, and India [4–6, 20]. There was little evidence of data analysis or system for monitoring the quality of data at any level. The district office simply acts as a relay station, which receives data from health facilities and forwards them to the Ministry of Health, where no database exists for generating trends and monitor thresholds of priority diseases for timely detection of incipient epidemics. Capacity building for routine data analysis, supportive supervision, sensitization, and motivation of surveillance staff at all levels as well as provision of logistic support and guidelines for data management and analysis at each level need to be prioritized [2, 20, 21].

Despite some achievements on epidemic preparedness and response in terms of structural arrangement, a central-level multi-sectoral emergency coordination committee, and rapid response teams for epidemics, substantial challenges remain. In the face of emerging public health threats and global pandemics, this function must be prioritized. Notably, epidemic preparedness plans should be evaluated and updated regularly; alert and epidemic threshold values as well as written guidelines and standard case management protocols for each epidemic prone disease should be prepared and used. It is equally important that functional mechanism of coordination and communication during suspected outbreaks needs to be strengthened [6, 18, 26, 30, 31]. In addition, risk assessment and inclusion of potential and emerging public health threats in the national/district epidemic preparedness plans need to be considered to ensure their recognition in the political agenda as suggested earlier [26].

This study found an inconsistent and inadequate feedback at all levels as previously reported for several other IDSR systems [2, 5, 8, 16, 20, 32, 33]. Neither health facilities nor districts had received formal written feedback reports except for periodic malaria bulletins and annual health bulletins produced by the Ministry on annual basis. Feedback is an essential function of any surveillance system and is an important component for promoting staff motivation and implementation of other IDSR functions [5, 16, 31].

Suboptimal performance of surveillance support functions undermines the performance of core functions [8, 16, 32]. Both supervision and training were weak as reported from elsewhere [2, 8]. Supervision was better at primary health facilities than hospitals and districts, the limitation attributed to higher staff workloads and insufficient transport facilities. Lack of clear guidelines or plans on the required number of supervisory visits made supervision less systematic and inefficient. Likewise, lack of training plans or database of trained surveillance personnel, competing priorities, financial resources largely affected provision of trainings in this study and in others [20, 30]. The fact that training was better at the district and national levels than at the health facility level has been reported by other studies [8, 20]. On-the-job trainings and refresher courses were sporadic and ad hoc, which resulted in very low knowledge on IDSR strategy and level-specific indicators among staff at all levels, which greatly hampered implementation of the strategy in general. Competent public health workforce is a key for strengthening performance of the IDSR strategy as well as planning and implementation of public health interventions for prevention and control of infectious diseases [10, 21, 29, 32–35]. To ensure sustainability, institutionalization of IDSR training into existing health training curricula is recommended [35].

Sustainable availability of resources is the basis of IDSR system performance [6, 30, 36]. In this study resources for surveillance activities were more available at national and district than at facility level. Major resource challenges at these higher levels were availability of communication services, and inadequate transportation capacities and funds, which hinder regular supervision, training, and outbreak investigation. Development of permanent cadre of skilled surveillance workforce particularly at the periphery/health facility, instead of relying on whoever becomes an in-charge, can enhance and sustain performance of the strategy [2, 20, 21]. To be able to execute their duties, trained surveillance personnel need to be equipped with adequate resources, job aids and logistic support for IDSR implementation [6, 10, 21, 22, 30].

Our observations align with the weaknesses expressed by surveillance staff at all healthcare system levels. The overarching challenges including financial constraints, insufficient staff training, and lack of electronic system for IDSR reporting all need urgent attention. Most importantly, for performance improvement and fostering commitment and accountability for the implementation of IDSR, sensitization and motivation of the implementing staff are crucial [15]. Motivation, defined as “an individual’s degree of willingness to exert and maintain an effort towards organizational goals” is fundamental for maintaining health system performance [37, 38]. Evidence indicate that, though essential, resource availability and staff competence are not sufficient to ensure high staff performance [37]. Vroom’s Expectancy Theory of motivation posit that, workers will be motivated to exert a high level of effort when they believe that they will be rewarded for the effort they put forth and the performance they achieve [39]. Health authorities in Zanzibar should make efforts to identify and establish appropriate incentive schemes for IDSR staff motivation.

It is to be noted that certain efforts to improve the IDSR strategy in Zanzibar are underway. The Ministry of health, with the support from Zanzibar WHO office, is currently finalizing revised IDSR guidelines, including an emphasis on community-based surveillance. Notably, following the conclusion of our data collection, computers have been procured for each public health facility and installation of an electronic IDSR system is reportedly in progress [Director of preventive services and health education, Ministry of Health Zanzibar, personal communication].

We acknowledge some limitations in our study. Firstly, this study did not include an assessment of surveillance quality attributes namely sensitivity, specificity, positive predictive values, representativeness, completeness, timeliness, flexibility, simplicity, usefulness, and acceptability of the system, which would add a broader understanding on performance and quality of the surveillance system. However, these attributes were beyond the objective and timeline of our study. Further study is needed to assess the quality of Zanzibar IDSR system. Secondly, a detailed laboratory assessment was not conducted, which would document the existing capacity for confirmation of each priority disease. Furthermore, only a small number of private facilities were included in this study. This is because, most people (> 70%) in Zanzibar prefer seeking care at public health facilities [40]. Lastly, this study did not employ a quantitative sample size calculation. Selection of health facilities was based on the WHO protocol for assessment of disease surveillance systems, which emphasized the importance of using a stratified sample that include all levels of surveillance systems, represent all geographical and administrative strata and health facilities to be randomly selected, which were all observed in this study [13].

## Conclusions

Despite some achievements including development of IDSR guidelines, integration of surveillance activities and structural organization, the current system is inadequate for early detection, reporting and responding to outbreaks and public health emergencies. The performance of both core and support surveillance functions are unsatisfactory and the surveillance workforce is inadequate and has limited capacity and motivation for IDSR operations. It is important that public health authorities should consider strengthening the IDSR strategy as a priority amid resource constraints. This evaluation provides important information for strengthening IDSR system performance, which is crucial for timely detection and response to infectious disease epidemics.

## Data Availability

All data generated or analysed during this study are included in this article. If someone wants to request the data from this study can contact the main author (FS).
